# Ubiquitin-specific protease 53 promotes osteogenic differentiation of human bone marrow-derived mesenchymal stem cells

**DOI:** 10.1038/s41419-021-03517-x

**Published:** 2021-03-04

**Authors:** Dawoon Baek, Kwang Hwan Park, Kyoung-Mi Lee, Sujin Jung, Soyeong Joung, Jihyun Kim, Jin Woo Lee

**Affiliations:** 1 Department of Orthopaedic Surgery, Yonsei University College of Medicine, 50-1 Yonsei-ro, Seodaemun-gu, Seoul 03722 South Korea; 2 Brain Korea 21 PLUS Project for Medical Sciences, Yonsei University College of Medicine, 50-1 Yonsei-ro, Seodaemun-gu, Seoul 03722 South Korea; 3 Severance Biomedical Science Institute, Yonsei University College of Medicine, 50-1 Yonsei-ro, Seodaemun-gu, Seoul 03722 South Korea

**Keywords:** Cell signalling, Mesenchymal stem cells

## Abstract

The ubiquitin protease pathway plays important role in human bone marrow-derived mesenchymal stem cell (hBMSC) differentiation, including osteogenesis. However, the function of deubiquitinating enzymes in osteogenic differentiation of hBMSCs remains poorly understood. In this study, we aimed to investigate the role of ubiquitin-specific protease 53 (USP53) in the osteogenic differentiation of hBMSCs. Based on re-analysis of the Gene Expression Omnibus database, USP53 was selected as a positive regulator of osteogenic differentiation in hBMSCs. Overexpression of USP53 by lentivirus enhanced osteogenesis in hBMSCs, whereas knockdown of USP53 by lentivirus inhibited osteogenesis in hBMSCs. In addition, USP53 overexpression increased the level of active β-catenin and enhanced the osteogenic differentiation of hBMSCs. This effect was reversed by the Wnt/β-catenin inhibitor DKK1. Mass spectrometry showed that USP53 interacted with F-box only protein 31 (FBXO31) to promote proteasomal degradation of β-catenin. Inhibition of the osteogenic differentiation of hBMSCs by FBXO31 was partially rescued by USP53 overexpression. Animal studies showed that hBMSCs with USP53 overexpression significantly promoted bone regeneration in mice with calvarial defects. These results suggested that USP53 may be a target for gene therapy for bone regeneration.

## Introduction

Bone homeostasis is regulated by the balance of osteogenesis (bone formation) and osteoclastogenesis (bone resorption)^1–3^. The imbalance of these processes leads to low bone density and deterioration of the bone matrix, which increases the risk of skeletal disorders, such as osteoporosis^4^. Osteoporosis causes serious socioeconomic problems^5,6^. Several therapeutics have been developed to maintain bone homeostasis^7,8^, including bone-forming and antiresorptive compounds. However, these compounds have serious side effects, including peptic ulcers, atypical femur fractures, osteonecrosis of the jaws, and increased risk of bone tumor^9,10^. Therefore, novel therapeutic agents are urgently needed.

The ubiquitin-proteasome pathway (UPP) is an ATP-dependent, reversible pathway that degrades target proteins^11^. Up to 80% of all intracellular proteins are degraded through the UPP. The reaction occurs through the hierarchal transfer of ubiquitin-activating enzyme (E1), ubiquitin-conjugating enzyme (E2), and ubiquitin ligase (E3), resulting in target protein degradation^11^. Deubiquitinating enzymes (DUBs) then remove ubiquitin chains from a target protein, controlling the function and stability of the protein^12,13^.

UPPs have important roles in the osteogenic differentiation of human bone marrow-derived mesenchymal stem cells (hBMSCs)^14^. Runt-related transcription factor 2 (RUNX2), a master transcription factor in osteogenesis, interacts with smad ubiquitination regulatory factor 1 (Smurf1) and Smurf2, E3 ligases, leading to the degradation of RUNX2^15^. As an E3 ligase, WW domain-containing E3 ubiquitin protein ligase 1 negatively regulates osteoblast differentiation targeting RUNX2^16^. The E3 ligase, Itch, also negatively regulates osteoblast function^17^. Accumulating evidence indicates that E3 ligases play important roles in the regulation of osteogenesis in hBMSCs^18,19^. However, few studies have addressed the functions of DUBs in the osteogenic differentiation of hBMSCs^20,21^.

Ubiquitin-specific peptidase 53 (USP53), a member of the deubiquitinating enzyme family, contains a catalytically inactive ubiquitin-specific protease domain and is expressed in cochlear hair cells^22^. Mice lacking *Usp*53 exhibit progressive hearing loss^22^; additionally, USP53 is mutated in novel syndromic forms of cholestatic liver disease in humans^23^. However, the functions of USP53 are unclear.

Accordingly, in this study, we identified USP53 as a differentially expressed gene (DEG) in re-analysis of the Gene Expression Omnibus (GEO) database and investigated the roles of USP53 in osteogenic differentiation of hBMSCs. We hypothesized that USP53 may positively regulate the osteogenic differentiation of hBMSCs in vitro and that local delivery of AAV2-USP53 to calvarial defects would thus enhance bone formation. Our findings provide important insights into the potential applications of AAV2-USP53-activated hBMSCs in gene therapy for bone regeneration.

## Results

### Expression of upregulated DUBs during the osteogenic differentiation of hBMSCs and selection of USP53 as a DUB-associated molecule

**Fig. 1:**
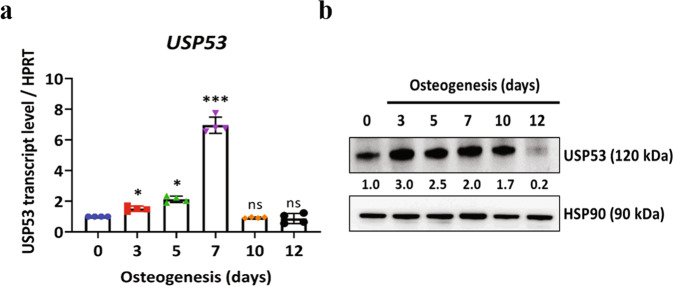
Upregulation of USP53 during the osteogenic differentiation of hBMSCs. **a** Relative mRNA expression levels of *USP53* during the osteogenic differentiation of hBMSCs. *HPRT* was used for normalization. **b** Protein levels of USP53 during the osteogenic differentiation of hBMSCs. Heat shock protein 90 (HSP90) was used as a loading control. USP53 protein expression was quantified using ImageJ. Day 0: undifferentiated hBMSCs. Results are means ± SDs. ns: not significant, *P < 0.05, ***P < 0.001 by one-way ANOVA. *n* = 3.

Based on GEO datasets and previous studies^24^, commonly upregulated DUBs in hBMSC osteogenesis were analyzed (Supplementary Table S1). From the selected DUBs, USP53 was found to show the most marked upregulation during the osteogenic differentiation of hBMSCs. Next, we examined the relative mRNA and protein levels of osteogenic markers during osteogenesis in hBMSCs. The mRNA and protein expression levels of osteogenic markers increased in the early phase of hBMSC osteogenesis (days 3–7) and decreased during the late phase of hBMSC osteogenesis (days 10–12; Supplementary Fig. 1). Consistent with the database findings, USP53 mRNA and protein expression levels were increased in the early phase and decreased in the late phase of osteogenesis in hBMSCs (Fig. 1a, b). Thus, USP53 may regulate the osteogenic differentiation of hBMSCs.

### Knockdown of USP53 inhibited the osteogenic differentiation of hBMSCs in vitro

**Fig. 2:**
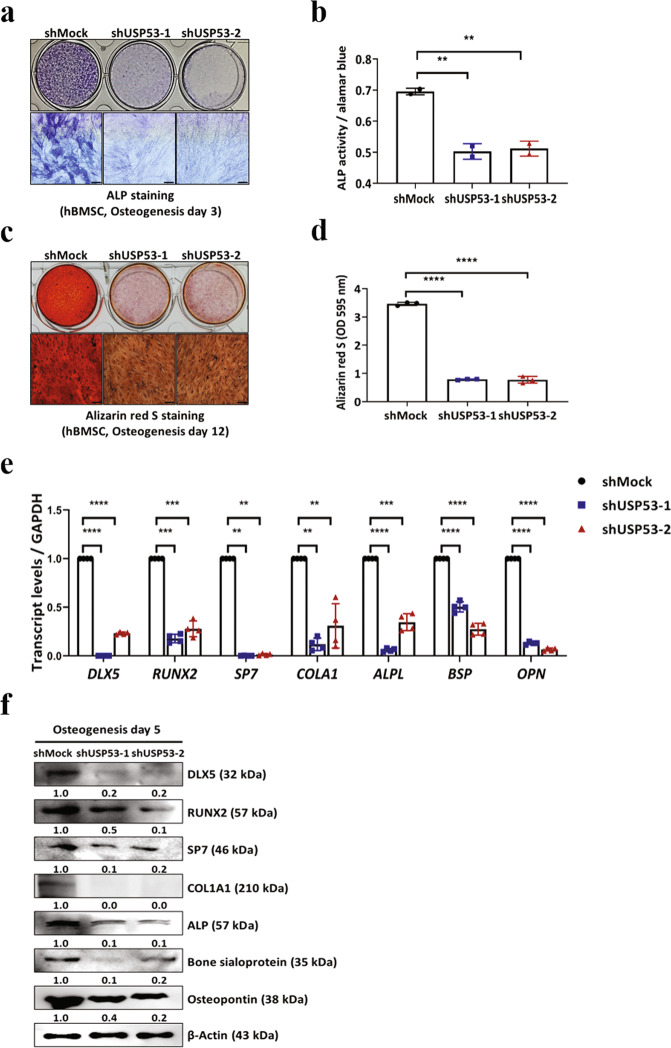
USP53 knockdown inhibits the osteogenic differentiation of hBMSCs in vitro. **a** Alkaline phosphatase staining was performed in shMock- or shUSP53-1-, 2-infected hBMSCs in osteogenic medium for 3 days. Scale bars, 200 μm. **b** Quantification of ALP activity. **c** Alizarin red S staining was performed in shMock- or shUSP53-1-, 2-infected hBMSCs in osteogenic medium for 12 days. Scale bars, 200 μm. **d** Quantification of Alizarin red S staining. **e** qRT-PCR analysis of osteogenesis-related genes on day 3 after osteogenic differentiation. **f** Immunoblot analysis of osteogenesis-related genes on day 5 after osteogenic differentiation. Data were quantified using ImageJ. Results are means ± SDs. ns: not significant, **P < 0.01, ***P < 0.001, ****P < 0.0001 by one-way ANOVA. *n* = 3.

We then investigated the roles of USP53 in the osteogenic differentiation of hBMSCs using short hairpin RNA (shRNA) targeting USP53. Approximately 90% knockdown efficiency was achieved (Supplementary Fig. 2a, b). Additionally, knockdown of USP53 decreased ALP and Alizarin Red S staining intensity as well as ALP activity (Fig. 2a–d). The effects of USP53 knockdown on osteogenic differentiation were not attributable to cell viability (Supplementary Fig. 3a, b). qRT-PCR and western blotting revealed that osteogenic marker levels in USP53-knockdown hBMSCs were decreased compared with that in shMock-transfected cells (Fig. 2e, f). These results suggested that knockdown of USP53 decreased the osteogenic differentiation of hBMSCs.

### Overexpression of USP53 promoted the osteogenic differentiation of hBMSCs in vitro

**Fig. 3:**
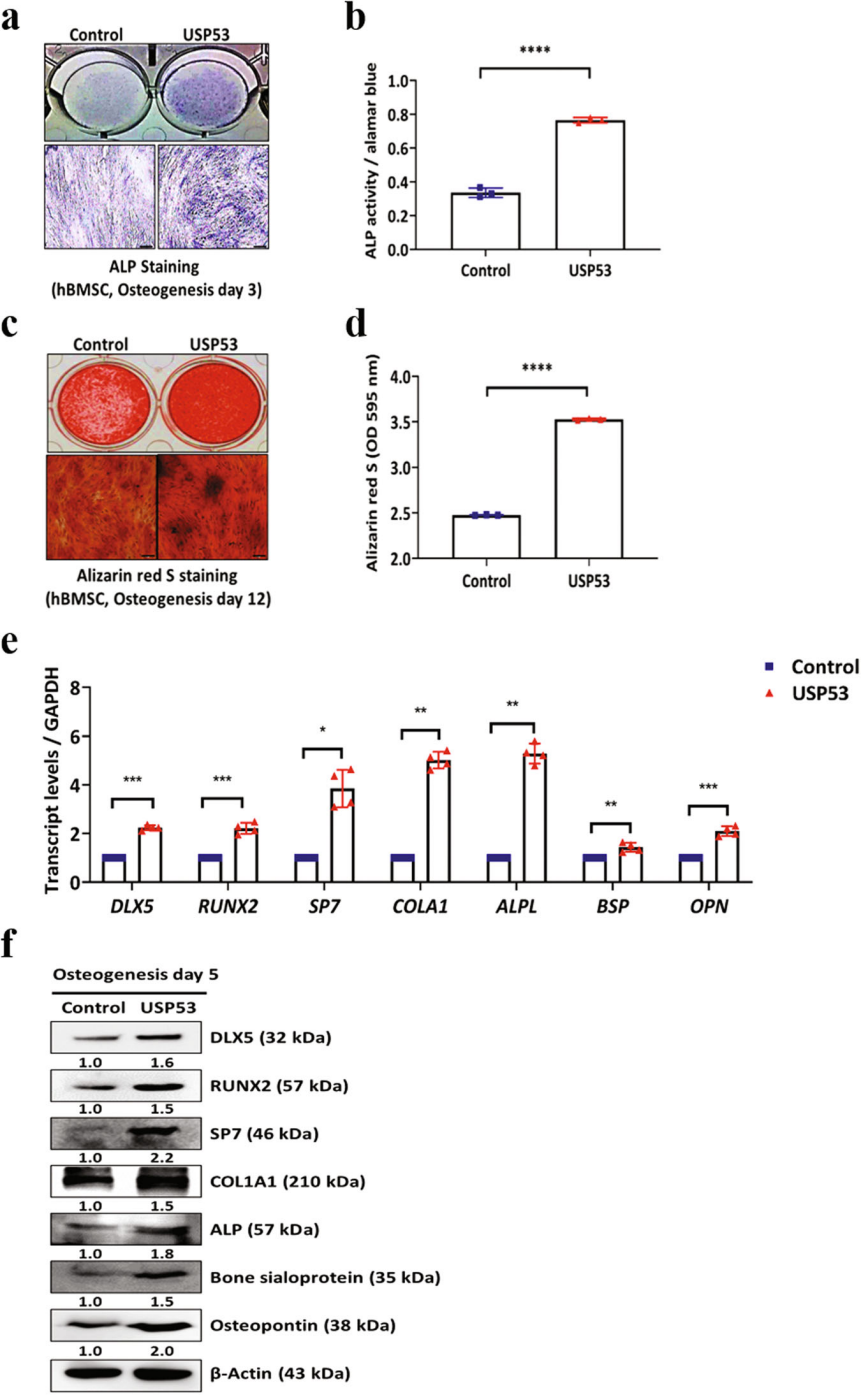
USP53 overexpression promotes the osteogenic differentiation of hBMSCs in vitro. **a** Alkaline phosphatase staining was performed in control or USP53-infected hBMSCs in osteogenic medium for 3 days. Scale bars, 200 μm. **b** Quantification of ALP activity. **c** Alizarin red S staining was performed in osteogenic medium for 12 days. Scale bars, 200 μm. **d** Quantification of Alizarin red S staining. **e** qRT-PCR analysis of osteogenesis-related genes on day 3 after osteogenic differentiation. **f** Immunoblot analysis of osteogenesis-related genes on day 5 after osteogenic differentiation. Data were quantified using ImageJ. Results are means ± SDs. *P < 0.05, **P < 0.01, ***P < 0.001, ****P < 0.0001 by unpaired two-tailed Student’s *t*-test. *n* = 3.

To further examine the roles of USP53 in the osteogenic differentiation of hBMSCs, we prepared lentiviral constructs expressing USP53 in hBMSCs. High overexpression efficiency was confirmed in USP53-overexpressing hBMSCs (Supplementary Fig. 2c, d). Overexpression of USP53 enhanced ALP and Alizarin red S staining intensity as well as ALP activity (Fig. 3a–d). The effects of overexpression of USP53 on osteogenic differentiation were not attributable to cell viability (Supplementary Fig. 3c, d). qRT-PCR and western blot analysis revealed that osteogenic marker expression levels in USP53-overexpressing hBMSCs were upregulated compared with those in control cells (Fig. 3e, f). Thus, USP53 positively regulated the osteogenic differentiation of hBMSCs.

### USP53 regulated the osteogenic differentiation of hBMSCs through Wnt/β-catenin signaling

**Fig. 4:**
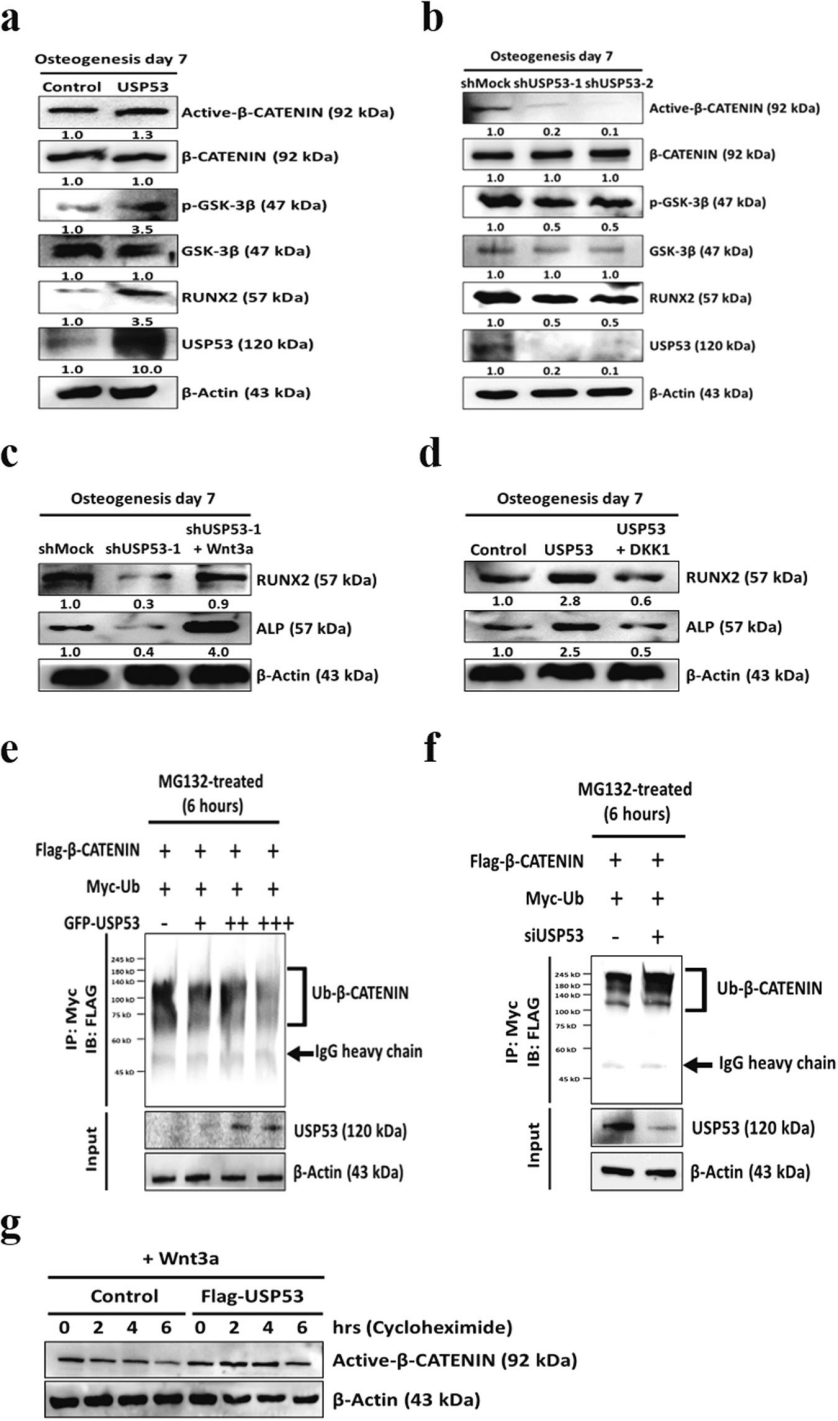
USP53 regulates osteogenic differentiation in hBMSCs through Wnt/β-catenin signaling. **a**, **b** Expression levels of Wnt/β-catenin signaling pathway mediators were investigated by immunoblotting in USP53-overexpressing (**a**) or -knockdown (**b**) hBMSCs in osteogenic medium for 7 days. Data were quantified using ImageJ. **c** Immunoblot analysis of Wnt3a-induced osteogenic marker expression. Data were quantified using ImageJ. **d** Immunoblot analysis of DKK1-induced osteogenic marker expression. Data were quantified using ImageJ. **e** Immunoblot analysis of β-catenin-linked polyubiquitination with overexpression of USP53. β-Catenin ubiquitination (top panel) and protein expression assays (bottom panel) were evaluated. **f** Immunoblot analysis of β-catenin-linked polyubiquitination with siUSP53. β-Catenin ubiquitination (top panel) and protein expression assays (bottom panel) were evaluated. **g** hBMSCs were transfected with Flag-β-catenin overexpression plasmid and control or Flag-USP53 and then treated with cycloheximide (100 μg/mL) and Wnt3a. Immunoblots with active β-catenin protein at the indicated time points. *n* = 3.

A signaling pathway involving Wnt/β-catenin, transforming growth factor-beta (TGF-β), bone morphogenetic protein (BMP), parathyroid hormone-related protein (PTHrP), and insulin-like growth factor (IGF) signaling plays important roles in regulating RUNX2 expression. Thus, western blot analysis was performed to evaluate the expression of Wnt/β-catenin signal pathway mediators in hBMSCs with overexpression or knockdown of USP53. The levels of unphosphorylated β-catenin were higher in hBMSCs overexpressing USP53 than in the control (Fig. 4a). Conversely, the levels of unphosphorylated β-catenin in USP53-knockdown hBMSCs were reduced compared with those in shMock-transfected cells (Fig. 4b). There were no significant differences in other signaling pathways (Supplementary Figs. 4 and 5 and Table S2).

To investigate whether USP53 promoted the osteogenesis of hBMSCs through the Wnt/β-catenin signaling pathway, we examined the effects of a Wnt/β-catenin activator (Wnt3a) and inhibitor (DKK1) in hBMSCs with knockdown or overexpression of USP53. Inhibition of the osteogenic differentiation capacity in the shUSP53 group was rescued in response to Wnt3a, an activator of the Wnt pathway (Fig. 4c). Conversely, enhanced osteogenic differentiation capacity in the USP53 overexpression group was reduced in response to DKK1, an inhibitor of the Wnt pathway (Fig. 4d), suggesting that USP53 may be essential for the osteogenic differentiation of hBMSCs through Wnt/β-catenin signaling. In vitro ubiquitination assays showed that β-catenin degradation was significantly reduced by USP53 overexpression in a concentration-dependent manner, whereas downregulation of USP53 promoted the degradation of β-catenin (Fig. 4e, f). Moreover, USP53 increased the half-life of active β-catenin compared with that in the control group (Fig. 4g). Taken together, these findings suggested that USP53 acted as a deubiquitinase enzyme, stabilizing β-catenin.

### Identification of USP53 binding proteins and regulation of β-catenin degradation by FBXO31 through the Skp1/Cul1/F-box protein (SCF) complex

**Fig. 5:**
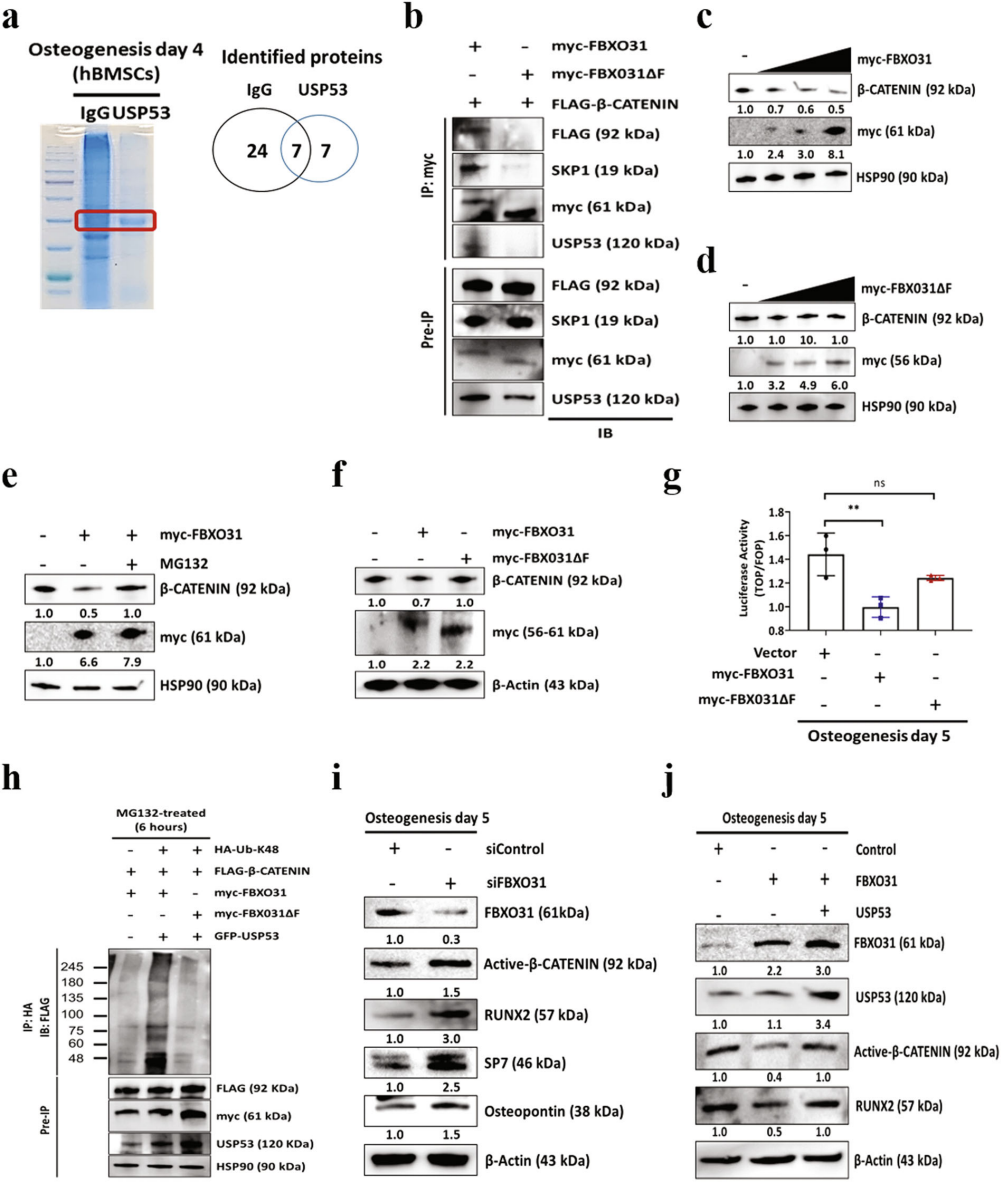
Identification of USP53 binding proteins and effects of FBXO31 on β-catenin degradation through the SCF complex. **a** Instantblue staining of a co-IP mixture separated by SDS-PAGE. The indicated band was extracted for analysis. **b** hBMSCs were cotransfected with FLAG-β-catenin with either myc-FBXO31 or myc-FBXO31ΔF for 48 h. Transfected cells were incubated with 10 μM MG132 for 6 h, and whole cells were lysed and subjected to IP with anti-myc antibodies. Immunoprecipitates and input protein extracts (Pre-IP) were resolved in SDS-PAGE. **c** hBMSCs were transfected with pCMV-myc or myc-FBXO31 for 48 h, and whole cell lysates were immunoblotted. **d** hBMSCs were transfected with pCMV-myc or myc-FBXO31ΔF for 48 h, and whole-cell lysates were immunoblotted. **e** hBMSCs were transfected with the pCMV-myc or myc-FBXO31 for 48 h. Transfected cells were then incubated with or without 10 μM MG132 for 6 h. Cell lysates were immunoblotted. **f** hBMSCs were transfected with pCMV-myc, myc-FBXO31, and myc-FBXO31ΔF for 48 h. Cells were harvested and lysed, and whole-cell protein extracts were immunoblotted. **g** β-Catenin transcriptional activity was measured on day 5 after induction of osteogenesis by TOP/FOP luciferase assays. **h** HEK293 cells were transfected with the indicated plasmids for 48 h, treated with 10 μM MG132 for 6 h, lysed, subjected to IP with anti-HA antibodies, and immunoblotted. **i** hBMSCs were transfected with negative control or FBXO31 siRNA for 5 days in osteogenic induction medium. Immunoblot analysis was performed, and data were quantified using ImageJ. **j** hBMSCs were transfected with empty vector, FBXO31, or GFP-USP53 and then cultured with osteogenic induction medium for 5 days. Immunoblot analysis was performed, and data were quantified using ImageJ. Results are means ± SDs. ns: not significant, **P < 0.01 by one-way ANOVA. *n* = 3.

Immunoprecipitation (IP) and LC–MS/MS were performed to identify proteins that interacted with USP53 in the osteogenesis of hBMSCs (Supplementary Table S3). Seven proteins were identified as interacting with USP53 during hBMSC osteogenesis, including F-box only protein 31 (FBXO31) (Fig. 5a). To examine whether USP53 interacted with FBXO31 in hBMSCs, co-IP and western blotting were performed using wild-type and mutant FBXO31 (myc-FBXO31ΔF), the latter of which harbored a deletion of the F-box domain, which mediates binding to Skp1 and Cul1. As shown in Fig. 5b, USP53 and FBXO31 bound with each other in hBMSCs. However, the FBXO31 mutant failed to interact with USP53, identifying FBXO31 as a novel binding partner of USP53.

FBXO31 is a candidate tumor-suppressor gene in breast, ovarian, hepatocellular, and prostate cancers^25^ and acts as a functional SCF-FBXO31 E3 ubiquitin ligase^25,26^. Therefore, we examined the effects of FBXO31 on the osteogenic differentiation of hBMSCs by controlling the expression of genes associated with osteogenesis. First, we examined the mechanism through which FBXO31 regulated the stability of β-catenin, a key mediator of the Wnt signaling pathway^27^ and bone formation during osteogenic differentiation^28^, using co-IP and western blot assays with wild-type FBXO31 and myc-FBXO31ΔF. As shown in Fig. 5b, FBXO31 specifically interacted with β-catenin, Skp1, and USP53. These results were effectively reversed by FBXO31 mutation (myc-FBXO31ΔF). Ectopic expression of FBXO31 or FBXO31 mutant at increasing concentrations in hBMSCs showed that FBXO31 significantly decreased β-catenin levels in a concentration-dependent manner; no changes were observed for the FBXO31 mutant (Fig. 5c, d). Moreover, FBXO31-mediated degradation of β-catenin was significantly blocked in the presence of MG132 (a 26S proteasome inhibitor), indicating that β-catenin stability was regulated at the post-translational level by FBXO31 (Fig. 5e).

F-box proteins bind with Skp1 to form an SCF complex through the F-box domain and bring their substrates to the complex for ubiquitination^29^. Therefore, to determine whether FBXO31 degraded β-catenin through the SCF complex, wild-type and mutant FBXO31 were overexpressed in hBMSCs. As shown in Fig. 5f, compared with wild-type FBXO31, FBXO31 mutant did not reduce β-catenin levels, indicating that FBXO31 degraded β-catenin through the SCF complex. Then, we examined whether FBXO31 regulated the transcriptional activity of β-catenin during the osteogenesis of hBMSCs using TOP/FOP assays. FBXO31 reduced the transcriptional activity of β-catenin during the osteogenesis of hBMSCs; however, no significant reduction was observed in the FBXO31 mutant during the same process (Fig. 5g). Finally, β-catenin polyubiquitination was confirmed by ubiquitination assays. FBXO31 mutant caused FBXO31 to lose the capability to promote β-catenin degradation (Fig. 5h). These results demonstrated that FBXO31 interacted with β-catenin and promoted the degradation of β-catenin through the SCF complex.

Next, we further investigated the effects of FBXO31 on the osteogenic differentiation of hBMSCs. First, we examined the effects of FBXO31 RNA interference on the osteogenic differentiation potential of hBMSCs. As shown in Fig. 5i, RNA interference of FBXO31 significantly increased active β-catenin and osteogenic marker levels compared with scrambled control siRNA. Conversely, FBXO31 overexpression decreased active β-catenin levels, resulting in reduced osteogenesis of hBMSCs. However, this phenomenon was rescued after modulating the expression of USP53 (Fig. 5j). These results demonstrated that the FBXO31-mediated reduction of osteogenesis in hBMSCs was significantly blocked in the presence of USP53.

### hBMSCs with AAV2-USP53 improved bone formation in vivo

**Fig. 6:**
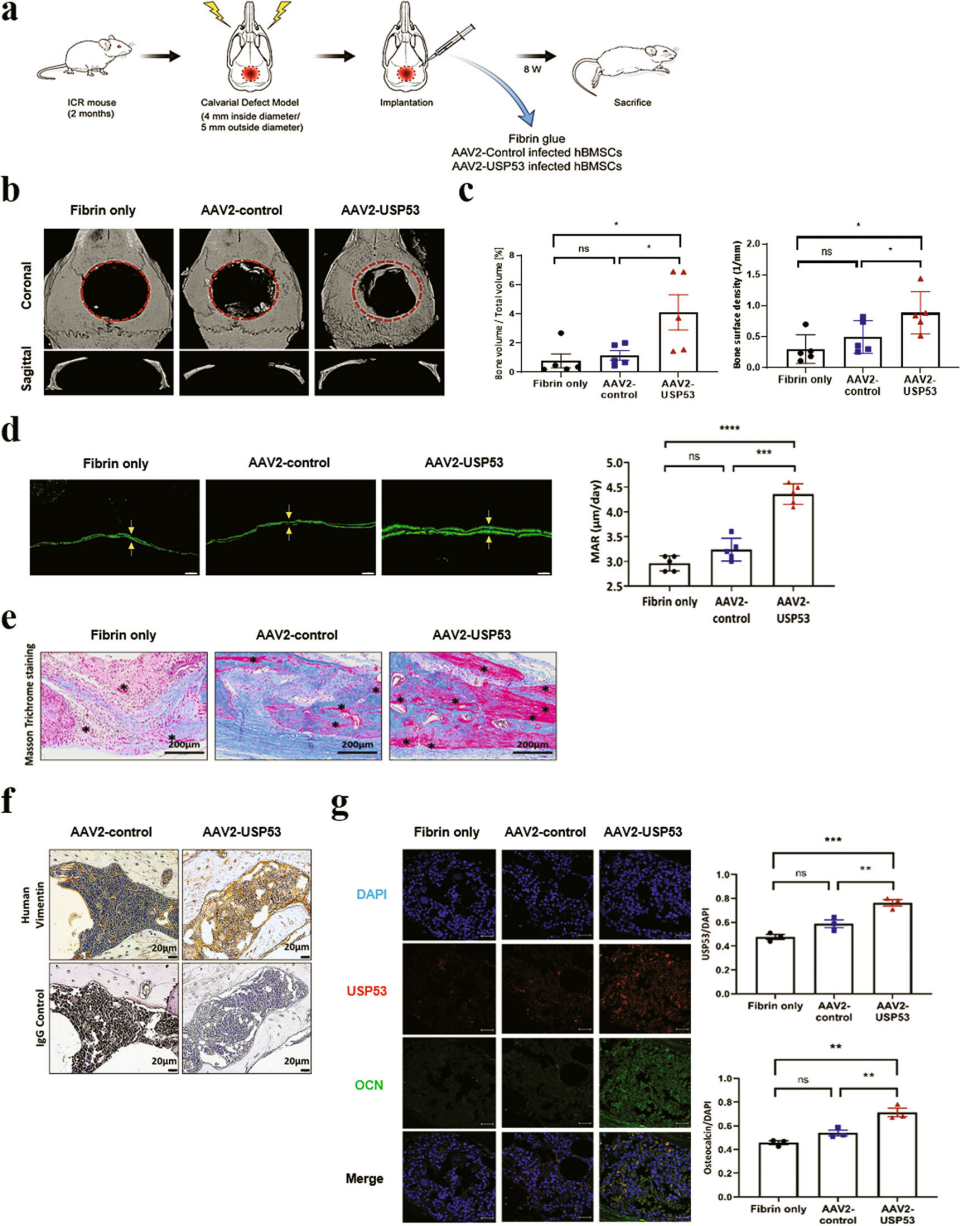
hBMSCs with AAV2-USP53 improved bone formation in vivo. **a** Experimental design of the mouse calvarial defect model. **b** Critical-size calvarial defects (5 mm in diameter) in mice were treated with AAV2-control-infected hBMSCs or AAV2-USP53-infected hBMSCs in fibrin matrix with PBS. Eight weeks after surgery, bone regeneration was measured by μCT. **c** Relative quantification of μCT analysis. **d** Histomorphometric analysis of calvarial defects in mice. Arrows indicate the distance between double calcein labeling. Scale bars, 20 μm. Relative histomorphometric quantification of the mineral apposition rate (MAR) is shown (right). **e** Representative images of M&T staining of calvarial bone sections in mice. Scale bars, 200 μm. *New bone. **f** Immunohistochemistry analysis using an antibody against human vimentin and IgG control of calvarial bone sections in mice. Scale bars, 20 μm. **g** Immunohistochemistry analysis of USP53 (phycoerythrin [PE]; red fluorescence) and OCN (fluorescein isothiocyanate [FITC]; green fluorescence) with DAPI counterstaining of the calvarial defects in mice (left). Quantification of IHC analysis (right). Scale bars, 50 μm. Results are presented as means ± SDs. ns: not significant, *P < 0.05, **P < 0.01, ***P < 0.001, ****P < 0.0001 by one-way ANOVA. *n* = 5 per group.

A mouse calvarial defect model was used to determine the effects of AAV2-USP53-infected hBMSCs on bone formation in vivo. Critical-size calvarial defects (5 mm in diameter) were created and then covered with fibrin glue mixed with AAV2-control-infected hBMSCs or AAV2-USP53-infected hBMSCs (Fig. 6a). Eight weeks after surgery, μCT images showed enhanced bone regeneration in the AAV2-USP53-infected hBMSC group compared with that in AAV2-control-infected hBMSCs (Fig. 6b). Quantification of μCT images showed that the proportion of bone volume per tissue volume (BV/TV) and bone surface density in the AAV2-USP53-infected hBMSC group were higher than those in the AAV2-control-infected hBMSC group (Fig. 6c). Calvarial surface calcein staining and mineral apposition rates were increased in the AAV2-USP53-infected hBMSC group (Fig. 6d).

Next, to investigate new bone formation, we performed M&T staining. The results showed that newly formed bone in the AAV2-USP53-infected hBMSC group was increased compared with that in the AAV2-control-infected hBMSC group (Fig. 6e). Evaluation of the expression of vimentin, a mesenchymal stem cell marker, by IHC showed that vimentin expression was increased in the AAV2-USP53-infected hBMSC group, indicating that the newly regenerated bone originated from the implanted hBMSCs (Fig. 6f). Next, we investigated the expression of osteocalcin (OCN), an important marker of extracellular matrix mineralization, by IHC analysis. The expression of OCN was higher in the AAV2-USP53-infected hBMSC group than in the AAV2-control-infected hBMSC group (Fig. 6g). Taken together, these results demonstrated that AAV2-USP53-infected hBMSCs had therapeutic effects on a calvarial defect murine model.

### USP53 expression was decreased in patients and mice with osteoporosis

**Fig. 7:**
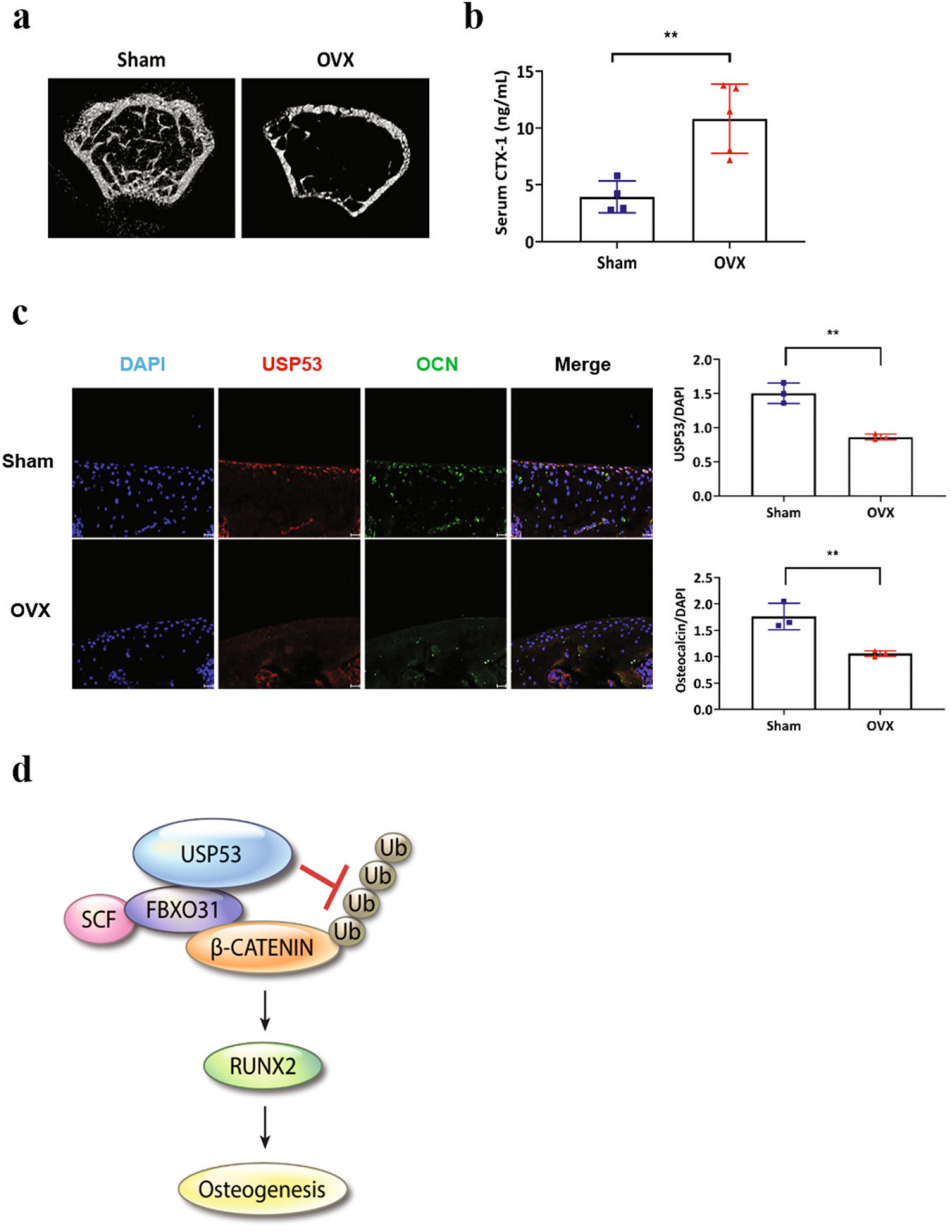
USP53 levels decrease in an osteoporosis mouse model. **a** Sham or ovariectomy (OVX) surgery was performed on 6-week-old female mice. Two months later, femoral trabecular bone mass was assessed by μCT. Scale bars, 50 μm. **b** Serum levels of the c-terminal telopeptide (CTX)-1 in sham and OVX mice. **c** Immunohistochemistry analysis of USP53 (phycoerythrin [PE]; red fluorescence) and OCN (fluorescein isothiocyanate [FITC]; green fluorescence) with DAPI counterstaining in sham-operated and OVX mice (left). Quantification of IHC analysis (right). Scale bars, 20 μm. Results are means ± SDs. **P < 0.01 by an unpaired two-tailed Student’s *t*-test. *n* = 5–10 per group. **d** Diagram showing the molecular mechanism through which FBXO31 and USP53 regulate the Wnt signaling mediator, β-catenin, during the osteogenic differentiation of hBMSCs.

Osteoporosis is a disease that causes bones to become weak without symptoms and increases the risk of broken bones. To explore the relationships between USP53 and osteoporosis, a GEO dataset (GSE35959)^30^ was analyzed. The results showed that USP53 expression in BMSCs from patients with osteoporosis was downregulated compared with that in normal controls (Supplementary Table S4). Furthermore, the roles of USP53 in osteoporosis were examined in an OVX mouse model to induce estrogen deficiency-related osteoporosis. μCT results showed that there was a significant reduction in trabecular bone mass in OVX mice compared with that in sham control mice (Fig. 7a). Next, we confirmed the expression of CTX-1 in OVX mice by ELISA. The expression of the bone resorption marker CTX-1 in OVX mice was increased compared with that in sham control mice (Fig. 7b). Moreover, the protein levels of USP53 in bone sections from OVX mice were also lower than those from sham mice, as confirmed by immunofluorescence staining (Fig. 7c). Taken together, these results demonstrated that USP53 expression was downregulated in patients and mice with osteoporosis.

## Discussion

Understanding the molecular mechanisms that regulate bone homeostasis is essential for the development of improved therapeutics to treat human skeletal disorders. UPPs are required for the osteogenic differentiation of hBMSCs^14^. Since USPs regulate cell cycle activity that is involved in post-translational modification, it is crucial in various diseases. Recent studies reported that USPs play an important role in cancer progression. In case of USP2a, it is overexpressed around 50% in human prostate cancers and affects the invasion ability of prostate cells^31^. USP22 is also upregulated in many cancer cells and activates the proliferation, migration, and invasion of gastric cancer, colorectal cancer, and breast cancer^32–34^. Another study reported that USP4 is overexpressed in hepatocellular carcinoma (HCC) tissues. And, USP4 increases progression of HCC through stabilization of cyclophilin A and deubiquitination^35^. Recently, USP34 has been reported as a positive regulator in osteogenic differentiation of hBMSCs^36^. Additionally, in the study about USP53, it was overexpressed in cervical cancer tissues and the inhibition of USP53 reduced cancer cell arrest^37^. But, none of the studies has reported about deubiquitinase enzyme which is related to osteoporosis. Here, we found that USP53 positively regulated the osteogenesis of hBMSCs in vitro and in vivo. Additionally, we confirmed that USP53 overexpression promoted osteogenesis in hBMSCs by activating the Wnt/β-catenin signaling pathway in vitro and that USP53 bound with FBXO31 during the osteogenic differentiation of hBMSCs. Mechanistically, FBXO31 negatively regulated the osteogenic differentiation of hBMSCs by acting as an E3 ubiquitin ligase and degrading β-catenin. Finally, USP53 expression was decreased in patients with osteoporosis and in a mouse model of estrogen deficiency-induced osteoporosis. Our findings provided further insights into the roles of DUBs in the pathogenesis of skeletal diseases and will be valuable for the development of novel therapeutic strategies for bone regeneration in tissue engineering.

We identified FBXO31 as a binding partner of USP53 and described its function in the osteogenesis of hBMSCs. F-box proteins (FBPs) are core components of E3 ubiquitin ligases and determine substrate specificity for degradation by protein targets^38^. Because the F-box motif is essential for the interactions within the SCF complex, this motif mediates the degradation of target proteins^39^. FBXO31 is a member of the FBP family and interacts with the SCF complex to form a functional SCF-FBXO31 E3 ubiquitin ligase, which regulates the ubiquitination of target proteins, such as mouse double minute 2, cyclin D1, forkhead box 1, chromatin licensing and DNA replication factor 1, Slug, mitogen-activated protein kinase kinase 6, and Par6c^26,40^. FBXO31 has been identified as a putative tumor-suppressor gene in breast, ovarian, hepatocellular, and prostate cancers. Its inactivation, due to heterozygosity loss, is associated with several cancers^41^. Additionally, FBXO31 has been reported to interact with the Skp1 protein^26^, and Cullin-1 and Roc-1 interact with myc-FBXO31/HA-Skp1^26^. Therefore, we hypothesized that FBXO31 may regulate the osteogenic differentiation of hBMSCs by modulating osteogenesis-related genes. Because the overexpression of USP53 by lentivirus results in enhanced osteogenic differentiation of hBMSCs through the Wnt/β-catenin signal pathway, β-catenin may be a target of the SCF-FBXO31 E3 ubiquitin ligase^42,43^. Here, we confirmed that SCF-FBXO31 bound with β-catenin and degraded β-catenin in hBMSCs. Moreover, FBXO31 mutant did not promoted the proteasomal degradation of β-catenin. Thus, FBXO31-SCF complex interaction was removed in FBXO31 mutant. Moreover, siFBXO31 enhanced the expression of active β-catenin and promoted the osteogenesis of hBMSCs. Conversely, overexpression of FBXO31 decreased active β-catenin and RUNX2 levels, thereby suppressing hBMSC osteogenesis. These results suggested that FBXO31 acted as an E3 ubiquitin ligase to degrade β-catenin, which then negatively regulated hBMSC osteogenesis. Accordingly, the FBXO31/β-catenin/USP53 axis may positively regulate the osteogenic differentiation of hBMSCs (Fig. 7d).

To date, AAV is considered the most promising vector for gene therapy. AAV is a nonenveloped parvovirus with a single-stranded DNA genome 4.7 kb in length. This genome encodes four nonstrucutural replication proteins (Rep78, Rep68, Rep52, and Rep40) and three structural capsid proteins (viral proteins 1–3)^44–46^. The most commonly used AAV serotype in bone biology is AAV2, which is a potential carrier for target gene delivery in the bone tissue^47–50^. Recently, AAV9 was classified as an attractive vector for therapeutic gene delivery in bone tissue^44,51,52^. In this study, we investigated the effects of USP53 overexpressed in hBMSCs by delivering AAV2 in a mouse calvarial defect model. These mice showed better regeneration, as characterized by increased BV/TV, bone surface density, and mineral apposition rates in comparison with control mice. However, marked bone regeneration was not observed in AAV2-USP53-infected hBMSCs. These results may be related to the complete removal of the periosteum, which has bone regeneration capacity.

We also analyzed the USP53 expression in hBMSCs of elderly patients (79–94 years old) with osteoporosis using a GEO dataset (GSE35959)^30^. Notably, USP53 expression in patients with osteoporosis was downregulated compared with that in normal controls. Similar results were observed in OVX mice. Thus, these findings indicated that USP53 may play important roles in the pathogenesis of bone disorders such as osteoporosis.

In summary, USP53 acted as a positive regulator of hBMSC osteogenic differentiation through Wnt/β-catenin signaling. The FBP, FBXO31, a component of the SCF complex, was shown to modulate the osteogenic differentiation of hBMSCs by acting as an E3 ubiquitin ligase to mediate the degradation of β-catenin. Intriguingly, USP53 was also shown to bind with the SCF ubiquitin ligase complex to reduce the polyubiquitination of β-catenin with FBXO31 dependently. Notably, transplantation of AAV2-USP53-infected hBMSCs improved bone regeneration in a mouse calvarial defect model. Taken together, our findings provide insights into the development of novel therapeutic strategies for the treatment of bone diseases. However, our findings are limited because we did not examine whether USP53 mutation/deletion was present in patients with osteoporosis. Further investigation is needed to determine the roles of USP53 in osteoblast lineages using *Prx1*-*Cre*;*Usp*53^f/f^, *Sp*7-*Cre*;*Usp*53^f/f^, or *Ocn*-*Cre*;*Usp*53^f/f^ mice.

## Materials and methods

### Cell culture and reagents

This study was approved by the Institutional Review Board of Yonsei University College of Medicine (IRB no. 4-2017-0232). Human bone marrow aspirates were obtained from the point 3 cm posterior to the anterior superior iliac spine of 10 healthy adult donors with a mean age of 35 years (range: 30–40 years). hBMSCs were maintained in low-glucose Dulbecco’s modified Eagle’s medium (DMEM-LG; Gibco BRL, Rockville, MD, USA) with 10% fetal bovine serum (FBS; Gibco) and 1% antibiotic-antimycotic solution (Gibco). Human embryonic kidney 293 (HEK293) and 293FT cells were obtained from American Type Culture Collection (Manassas, VA, USA). These cells were maintained in high-glucose DMEM (DMEM-HG; Gibco) containing 10% FBS and 1% antibiotic-antimycotic solution (Gibco). DKK1 (R&D Systems) was used at a concentration of 0.1 mg/mL in hBMSCs^53^. Wnt3a (cat. no. 5036-WN; R&D Systems) was used at a concentration of 10 ng/mL in hBMSCs^54,55^.

### Microarray re-analysis

The mRNA expression profiles of hBMSCs in osteogenic differentiation medium were found in GEO datasets (https://www.ncbi.nlm.nih.gov/geo/) based on GSE18043^24^. A web-based tool, GEO2R (http://www.ncbi.nlm.nih.gov/geo/geo2r/) was used to analyze the DEGs by comparing up-regulated DUBs during hBMSC osteogenesis at different time points (day 0 versus days 1, 3, or 7). The cut-off value was set as *P* < 0.05, log (fold change) > 2.0. The mRNA expression profiles of hBMSCs from five patients with osteoporosis (79–94 years old) were found in GEO datasets based on GSE35959^30^ that contained a total of 9 samples (hMSCs derived from 3 normal females, 1 normal male donor, and 5 osteoporosis female patients). The cut-off value was set as *P* < 0.05, log (fold change) < 0.5.

### Osteogenic differentiation of hBMSCs

hBMSCs were seeded onto 12-well culture plates at a density of 8 × 10^4^ cells/well and then maintained for 10 days in osteogenic medium (DMEM-LG containing 10% FBS, 1% antibiotic-antimycotic solution, 100 nM dexamethasone, 10 mM β-glycerophosphate, and 50 μg/mL ascorbic acid)^53^. The osteogenic medium was replenished every 3 days. Alizarin red S and alkaline phosphatase (ALP) staining procedures were performed as previously described^44^.

### Quantitative real-time reverse transcription polymerase chain reaction (qRT-PCR)

Total RNA was isolated from cells using an RNeasy kit (cat. no. 74106; Qiagen, Valencia, CA, USA). RNA (2 μg) was then reverse transcribed using an Omniscript kit (cat. no. 205113; Qiagen). qRT-PCR was performed using a StepOnePlus Real-Time PCR System (Applied Biosystems, Foster City, CA, USA) and 2× qPCRBIO SyGreen Mix (cat. no. PB20.12-05; PCR Biosystems) according to the manufacturers’ guidelines. Primer sequences are listed in Supplementary Table S5.

### Western blotting

Total proteins were prepared from cells using passive lysis buffer (cat. no. E194A; Promega, Madison, WI, USA), and 30 μg of each sample was separated by sodium dodecyl sulfate polyacrylamide gel electrophoresis (SDS-PAGE) and transferred to polyvinylidene difluoride membranes. After blocking with tris-buffered saline with Tween 20 containing 5% skim milk, blots were incubated overnight at 4 °C with primary antibodies. All membranes were then incubated with horseradish peroxidase-conjugated secondary antibodies (1:5000; GenDEPOT) for 1 h at room temperature. Finally, the western blots were imaged using LAS 4000 (Fujifilm). Primary antibodies are listed in Supplementary Table S6.

### Plasmids and RNA interference

FLAG-HA-USP53 was gifted by Wade Harper (Addgene plasmid #22606). FLAG-β-catenin was a gift from Eric Fearon (Addgene plasmid #16828). pCMV-Myc (Clontech plasmid #631604) was purchased from Clontech (TaKaRa, Shiga, Japan). myc-F-box only protein 31 (FBXO31) and myc-FBXO31ΔF were kind gifts from Prof. David F. Callen (University of Adelaide and Hanson Institute, Adelaide, Australia). The FBOX31 clone (untagged) was provided by the Korea Human Gene Bank, Medical Genomics Research Center (KRIBB, Daejeon, South Korea). Green fluorescent protein (GFP)-RUNX2, myc-Ub, HA-Ub, and HA-Ub(K48) were kindly provided by Prof. Jae-Hyuck Shim (University of Massachusetts Medical School, Worcester, MA, USA). Lentiviral control vector (pLenti-GIII-CMV-GFP-2A-Puro, LV590) and lentiviral vector-USP53 (pLenti-GIII-CMV-USP53-GFP-2A-Puro, LV353779) were purchased from Applied Biological Materials (Richmond, BC, Canada). Scramble negative control small interfering RNA (siRNA; siRNA no. SN1002), USP53 siRNA (siRNA no. 54532), and FBXO31 siRNA (siRNA no. 79791) were purchased from Bioneer (Daejeon, South Korea).

### Lentivirus production

293FT cells were transfected with USP53-overexpressing lentivirus or USP53 shRNA along with packaging plasmid (delta 8.9) and envelop plasmid (VSV-G) using Lipofectamine LTX & Plus Reagent (Invitrogen, Carlsbad, CA, USA) according to the manufacturer’s instructions. DMEM-LG supplemented with 10% FBS was changed 6 h after transfection. The supernatants containing virus were collected 24 and 48 h after initial transfection, filtered with a 0.45-μm syringe filter (Millipore), separated into aliquots, and stored at -80 °C until use. hBMSCs were seeded onto 6-well plates at a density of 1 × 10^5^ cells/well and transfected with purified lentiviruses for 24 h. Puromycin (10 μg/mL; Sigma, St. Louis, MO, USA) was used for selection, and stable lentivirus-infected hBMSCs were used in experiments. Information on the lentiviral vectors is provided in Supplementary Table S7.

### Adeno-associated virus transduction

AAV blank control with GFP (serotype 2, 10^9^ genome copies [GC]/mL, AAV2-control, AAVP022) and AAV-USP53 with GFP (serotype 2, 10^9^ GC/mL, AAV2-USP53, AAVP4915843) were purchased from Applied Biological Materials. For AAV infection, hBMSCs were seeded onto 12-well plates at a density of 8 × 10^4^ cells/well. After 24 h, hBMSCs were incubated with 40 μL AAV2-control and AAV2-USP53 using X-tremeGENE^TM^9 DNA transfection reagent (Sigma). AAVs infected with hBMSCs were grown to 80% confluence and then replated at a density of 1 × 10^5^ cells in a 10-cm^2^ dish.

### Immunoprecipitation analysis

Transfected cells were incubated with 10 μM MG132 (cat. no. M7749; Sigma) for 6 h, and whole cells were lysed using nondenaturing lysis buffer (20 mM Tris-HCl [pH 8.0], 137 mM NaCl, 0.5% Nonidet P40 with 2 mM ethylenediaminetetraacetic acid [EDTA, pH 8.0], 1 mM phenylmethylsulfonyl fluoride, and protease inhibitor). The lysates were incubated with anti-myc or HA antibodies overnight at 4 °C. Then, lysates were conjugated with 40 μL pre-washed protein A/G agarose beads (Santa Cruz Biotechnology, Santa Cruz, CA, USA) for 4 h at 4 °C with gentle rotation. Immunoprecipitates were eluted with 2× lane marker-reducing sample buffer (Thermo Fisher Scientific). Eluates were separated using SDS-PAGE.

### Liquid chromatography tandem mass spectrometry (LC-MS/MS)

To investigate USP53-binding proteins in hBMSCs, the cells were transfected with lentiviral control vector or lentiviral vector-USP53 and cultured in osteogenic induction medium for 4 days. The cells were then treated with MG132 (10 μM) for 6 h, and hBMSCs were harvested using passive lysis buffer (Promega). Total protein (approximately 500 μg) was incubated with an antibody against GFP (cat. no. sc-390394; Santa Cruz Biotechnology) and IgG control (cat. no. 12-370; Milipore) at 4 °C overnight. SDS-PAGE was performed, following which the gels were stained with an InstantBlue staining kit (cat. no. ISB1L; Sigma). Differential bands were collected for LC-MS/MS, which was performed using a LTQ orbitrap mass spectrometer (Thermo Electron, San Jose, CA, USA).

### Luciferase reporter assays

hBMSCs were seeded onto 6-well plates at a density of 1 × 10^5^ cells/well, and cells were transfected with 1 μg pCMV-myc, myc-FBXO31, myc-FBXO31ΔF plus TOP-flash (wild-type T cell factor binding site), or FOP-flash (mutant T cell factor binding site) using Lipofectamine LTX & Plus Reagent (Invitrogen) according to the manufacturer’s instructions. After 24 h, the cells were maintained for 5 days in osteogenic induction medium. The cells were analyzed using a simple dual-luciferase assay (Promega).

### Protein degradation assay

hBMSCs were seeded at a density of 1 × 10^5^ cells/well onto 6-well plates and transfected with Flag-β-catenin and control or Flag-USP53. Cells were cultured in the presence of Wnt3a. After 48 h, 100 μg/mL cycloheximide (cat. no. C4859; Sigma) was added. Cells were harvested at the indicated time points.

### Animal experiments

The Committee on the Ethics of Animal Experiments of Yonsei University College of Medicine approved all animal experiments and protocols (permit no. IACUC-2019-0168). Fifteen male Institute of Cancer Research (ICR) mice (8 weeks old) were anesthetized with zoletil (30 mg/kg body weight; Virbac Laboratories) and rompun (10 mg/kg body weight; Bayer Korea) by intraperitoneal injection. After shaving the skull, an alcohol swab was used before hair trimming. The skin was retracted. Critical-sized calvarial bone defects (4 mm inside diameter/5 mm outside diameter) were created using a trephine burr (Shudent). The animals were divided into three groups as follows: (1) fibrin gel only, (2) hBMSCs (5 × 10^5^) with AAV2-control group, and (3) hBMSCs (5 × 10^5^) with AAV2-USP53. Fibrin gel (20 μL; TISSEEL) was mixed with hBMSCs and implanted into defects. Finally, the defect site was closed with vicryl plus (Ethicon 3-0) and nylon (Dermalon 3-0). Metacam (1 mg/kg) was used as an analgesic. Mice were sacrificed 8 weeks after surgery, and the skulls were harvested.

### Micro-computed tomography (μCT) and histological analysis

Murine skulls were fixed in 4% paraformaldehyde (PFA) for 3 days, following which μCT was performed using a Skyscan 1173 High Energy Micro-CT scanner (Skyscan NV, Kontich, Belgium) with a 40–130 kV, 8 W X-ray source. For histological analysis, skulls or femurs were decalcified with 5% formic acid (Sigma) or 0.5 M EDTA for 1 week. Fixed specimens were then embedded in paraffin blocks and cut into 4-μm-thick sections. Sections were deparaffinized in xylene and serially rehydrated in ethanol. Sections were sequentially stained with Masson trichrome (M&T) staining and immunohistochemistry (IHC). Primary antibodies are listed in Supplementary Table S8. For, 3,3′-diaminobenzidine tetrahydrochloride staining, we used a Dako REAL EnVision Detection System (cat. no. K5007; DAKO), and counterstaining was performed with hematoxylin (cat. no. GHS-3; Sigma). A Zeiss LSM 700 confocal laser scanning microscope (Carl Zeiss Micro Imaging GmbH, Jena, Germany) was used to visualize images. For performing mouse calvarial bone histomorphometry, 25 mg/kg calcein (cat. no. C0875; Sigma) dissolved in 2% sodium bicarbonate solution was subcutaneously injected into mice at 4-day intervals (days 6 and 2 before sacrifice). Undecalcified skull samples were fixed in 4% PFA for 3 days and then embedded in plastic resin blocks (methyl methacrylate) for obtaining bone sections (7 μm).

### Enzyme-linked immunosorbent assay (ELISA)

The Committee on the Ethics of Animal Experiments of Yonsei University College of Medicine approved all animal experiments and protocols (permit no. IACUC-2020-0045). Female sham-operated and ovariectomized (OVX) mice (C57BL/6J, 6 weeks old) were purchased from Japan SLC (Hamamatsu, Japan). After 8 weeks, the sham and OVX mice were sacrificed, and serum was collected by cardiac puncture. Mouse RatLaps (C-terminal telopeptide type I collagen [CTX-1]; cat. no. AC-06F1; IDS) was used according to the manufacturer’s recommendations to measure serum level of CTX-1.

### Statistical analysis

For each experiment, samples were analyzed at least in triplicate. Results with *P* values less than 0.05 were considered statistically significant. All data are presented as means ± standard deviations (SDs). No animals or samples were excluded from the analysis. We first performed Shapiro-Wilk normality tests for normal distributions of the groups. If normality tests were passed, two-tailed, unpaired Student’s t-tests were used for the comparisons between two groups. For comparisons of three or four groups, we used one-way analysis of variance (ANOVA) if normality tests were passed, followed by Tukey’s multiple comparison tests for all pairs of groups. GraphPad PRISM software (version 8.0; La Jolla, CA, USA) was used for statistical analysis.

## Supplementary information

Supplementary Table S1–S8

Supplementary Figure 1

Supplementary Figure 2

Supplementary Figure 3

Supplementary Figure 4

Supplementary Figure 5

## References

[1] Bernard NJ (2019). Sensing bone mass. Nat. Rev. Rheumatol..

[2] Ray K (2014). Bone: The immune system takes control of bone homeostasis. Nat. Rev. Rheumatol..

[3] Modinger Y, Loffler B, Huber-Lang M, Ignatius A (2018). Complement involvement in bone homeostasis and bone disorders. Semin Immunol..

[4] Harada S, Rodan GA (2003). Control of osteoblast function and regulation of bone mass. Nature.

[5] Eastell R (2016). Postmenopausal osteoporosis. Nat. Rev. Dis. Primers..

[6] Morris JA (2019). An atlas of genetic influences on osteoporosis in humans and mice. Nat. Genet..

[7] Hopkins RB (2011). The relative efficacy of nine osteoporosis medications for reducing the rate of fractures in post-menopausal women. BMC Musculoskelet. Disord..

[8] Khosla S, Hofbauer LC (2017). Osteoporosis treatment: recent developments and ongoing challenges. Lancet Diabetes Endocrinol..

[9] Coleman RE (2008). Risks and benefits of bisphosphonates. Br. J. Cancer.

[10] Borumandi F, Aghaloo T, Cascarini L, Gaggl A, Fasanmade K (2015). Anti-resorptive drugs and their impact on maxillofacial bone among cancer patients. Anti-Cancer Agent Me.

[11] Lecker SH, Goldberg AL, Mitch WE (2006). Protein degradation by the ubiquitin-proteasome pathway in normal and disease states. J. Am. Soc. Nephrol..

[12] Mevissen TET, Komander D (2017). Mechanisms of deubiquitinase specificity and regulation. Annu. Rev. Biochem..

[13] Harrigan JA, Jacq X, Martin NM, Jackson SP (2018). Deubiquitylating enzymes and drug discovery: emerging opportunities. Nat. Rev. Drug Disco..

[14] Severe N, Dieudonne FX, Marie PJ (2013). E3 ubiquitin ligase-mediated regulation of bone formation and tumorigenesis. Cell Death Dis..

[15] Kaneki H (2006). Tumor necrosis factor promotes Runx2 degradation through up-regulation of Smurf1 and Smurf2 in osteoblasts. J. Biol. Chem..

[16] Shu L, Zhang H, Boyce BF, Xing L (2013). Ubiquitin E3 ligase Wwp1 negatively regulates osteoblast function by inhibiting osteoblast differentiation and migration. J. Bone Min. Res..

[17] Liu J (2017). Ubiquitin E3 ligase Itch negatively regulates osteoblast function by promoting proteasome degradation of osteogenic proteins. Bone Jt. Res.

[18] Thacker G (2016). Skp2 inhibits osteogenesis by promoting ubiquitin-proteasome degradation of Runx2. Biochim Biophys. Acta.

[19] Shim JH (2013). Schnurri-3 regulates ERK downstream of WNT signaling in osteoblasts. J. Clin. Invest..

[20] Tang YM (2017). Protein deubiquitinase USP7 is required for osteogenic differentiation of human adipose-derived stem cells. Stem Cell Res..

[21] Guo YC (2018). Ubiquitin-specific protease USP34 controls osteogenic differentiation and bone formation by regulating BMP2 signaling. Embo J..

[22] Kazmierczak M (2015). Progressive hearing loss in mice carrying a mutation in Usp53. J. Neurosci..

[23] Maddirevula S (2019). Identification of novel loci for pediatric cholestatic liver disease defined by KIF12, PPM1F, USP53, LSR, and WDR83OS pathogenic variants. Genet. Med..

[24] Hamidouche Z (2009). Priming integrin alpha5 promotes human mesenchymal stromal cell osteoblast differentiation and osteogenesis. Proc. Natl Acad. Sci. USA.

[25] Johansson P (2014). SCF-FBXO31 E3 ligase targets DNA replication factor Cdt1 for proteolysis in the G2 phase of cell cycle to prevent re-replication. J. Biol. Chem..

[26] Kumar R (2005). FBXO31 is the chromosome 16q24.3 senescence gene, a candidate breast tumor suppressor, and a component of an SCF complex. Cancer Res..

[27] Kramer I (2010). Osteocyte Wnt/beta-catenin signaling is required for normal bone homeostasis. Mol. Cell Biol..

[28] Gaur T (2005). Canonical WNT signaling promotes osteogenesis by directly stimulating Runx2 gene expression. J. Biol. Chem..

[29] Paul D (2019). F-box protein FBXO16 functions as a tumor suppressor by attenuating nuclear beta-catenin function. J. Pathol..

[30] Benisch P (2012). The transcriptional profile of mesenchymal stem cell populations in primary osteoporosis is distinct and shows overexpression of osteogenic inhibitors. PLoS ONE..

[31] Benassi B (2012). MYC is activated by USP2a-mediated modulation of microRNAs in prostate cancer. Cancer Discov..

[32] Ma Y (2017). USP22 maintains gastric cancer stem cell stemness and promotes gastric cancer progression by stabilizing BMI1 protein. Oncotarget.

[33] Kim D (2017). Deubiquitinating enzyme USP22 positively regulates c-Myc stability and tumorigenic activity in mammalian and breast cancer cells. J. Cell. Physiol..

[34] Ji M (2015). Ubiquitin specific protease 22 promotes cell proliferation and tumor growth of epithelial ovarian cancer through synergy with transforming growth factor β1. Oncol. Rep..

[35] Li T (2018). Ubiquitin-specific protease 4 promotes hepatocellular carcinoma progression via cyclophilin A stabilization and deubiquitination. Cell death Dis..

[36] Pathria G (2018). Targeting the Warburg effect via LDHA inhibition engages ATF4 signaling for cancer cell survival. EMBO J..

[37] Zhou Q, Yao X, Wu C, Chen S, Fan D (2019). Knockdown of ubiquitin-specific protease 53 enhances the radiosensitivity of human cervical squamous cell carcinoma by regulating DNA damage-binding protein 2. Technol. Cancer Res. Treat..

[38] Gong J, Huo JR (2015). New insights into the mechanism of F-box proteins in colorectal cancer (Review). Oncol. Rep..

[39] Malonia SK, Dutta P, Santra MK, Green MR (2015). F-box protein FBXO31 directs degradation of MDM2 to facilitate p53-mediated growth arrest following genotoxic stress. P Natl Acad. Sci. USA.

[40] Tan Y, Liu D, Gong J, Liu J, Huo J (2018). The role of F-box only protein 31 in cancer. Oncol. Lett..

[41] Dutta P (2019). The tumor suppressor FBXO31 preserves genomic integrity by regulating DNA replication and segregation through precise control of cyclin A levels. J. Biol. Chem..

[42] Kimelman D, Xu W (2006). beta-catenin destruction complex: insights and questions from a structural perspective. Oncogene.

[43] Su Y, Ishikawa S, Kojima M, Liu B (2003). Eradication of pathogenic beta-catenin by Skp1/Cullin/F box ubiquitination machinery. Proc. Natl Acad. Sci. USA.

[44] Yang YS (2019). Bone-targeting AAV-mediated silencing of Schnurri-3 prevents bone loss in osteoporosis. Nat Commun..

[45] Gao G, Vandenberghe LH, Wilson JM (2005). New recombinant serotypes of AAV vectors. Curr. Gene Ther..

[46] Becerra SP, Koczot F, Fabisch P, Rose JA (1988). Synthesis of adeno-associated virus structural proteins requires both alternative messenger-rna splicing and alternative initiations from a single transcript. J. Virol..

[47] Chen Y (2003). Gene therapy for new bone formation using adeno-associated viral bone morphogenetic protein-2 vectors. Gene Ther..

[48] Kang Y (2007). In vitro and in vivo induction of bone formation based on adeno-associated virus-mediated BMP-7 gene therapy using human adipose-derived mesenchymal stem cells (vol 28, pg 839, 2007). Acta Pharm. Sin..

[49] Gafni Y (2004). Gene therapy platform for bone regeneration using an exogenously regulated, AAV-2-based gene expression system. Mol. Ther..

[50] Choi VW, McCarty DM, Samulski RJ (2005). AAV hybrid serotypes: improved vectors for gene delivery. Curr. Gene Ther..

[51] Lee LR (2019). Targeting Adeno-Associated Virus Vectors for Local Delivery to Fractures and Systemic Delivery to the Skeleton. Mol. Ther. Methods Clin. Dev..

[52] Luo FT (2018). Adeno-Associated Virus-Mediated RNAi against Mutant Alleles Attenuates Abnormal Calvarial Phenotypes in an Apert Syndrome Mouse Model. Mol. Ther.-Nucl. Acids.

[53] Park KH (2018). Zinc Promotes Osteoblast Differentiation in Human Mesenchymal Stem Cells Via Activation of the cAMP-PKA-CREB Signaling Pathway. Stem cells Dev..

[54] Lai WT, Krishnappa V, Phinney DG (2011). Fibroblast growth factor 2 (Fgf2) inhibits differentiation of mesenchymal stem cells by inducing Twist2 and Spry4, blocking extracellular regulated kinase activation, and altering Fgf receptor expression levels. Stem cells (Dayt., Ohio).

[55] Kim JA, Choi HK, Kim TM, Leem SH, Oh IH (2015). Regulation of mesenchymal stromal cells through fine tuning of canonical Wnt signaling. Stem cell Res..

