# Supplementary material for: Ubiquitin-specific protease 53 promotes osteogenic differentiation of human bone marrow-derived mesenchymal stem cells
Source: Cell Death Dis. 2021 Mar 4;12(3):238. doi: 10.1038/s41419-021-03517-x (PMC7933275; doi:10.1038/s41419-021-03517-x)
Supplement: Supplementary file 1 — Supplementary Table S1–S8 [file 41419_2021_3517_MOESM1_ESM.docx]

**Supplementary Tables**

**Ubiquitin-specific protease 53 promotes osteogenic differentiation of human bone marrow-derived mesenchymal stem cells**

Dawoon Baek^1,2,4^, Kwang Hwan Park^1,4^, Kyoung-Mi Lee^1,3^, Sujin Jung^1,2^, Soyeong Joung^1,2^, Jihyun Kim^1,2^, Jin Woo Lee*^1,2,3^

^1^Department of Orthopaedic Surgery, Yonsei University College of Medicine, 50-1 Yonsei-ro, Seodaemun-gu, Seoul 03722, South Korea

^2^Brain Korea 21 PLUS Project for Medical Sciences, Yonsei University College of Medicine, 50-1 Yonsei-ro, Seodaemun-gu, Seoul 03722, South Korea

^3^Severance Biomedical Science Institute, Yonsei University College of Medicine, 50-1 Yonsei-ro, Seodaemun-gu, Seoul 03722, South Korea

These authors contributed equally: Dawoon Baek, Kwang Hwan Park

**Running title**: Ubiquitin-specific protease 53 and osteogenesis

**Conflict of interest:** The authors declare no competing interests.

***Corresponding author** JWL (E-mail: ljwos@yuhs.ac)

**Supplementary Table S1. Upregulated deubiquitinating enzymes (DUBs) in osteogenesis, as determined by analysis of microarray data.**

| **Up-regulated DUBs**  (day 1 versus day 0)  **(*P* < 0.05)** | | **Up-regulated DUBs**  (day 3 versus day 0)  **(*P* < 0.05)** | | **Up-regulated DUBs**  (day 7 versus day 0)  **(*P* < 0.05)** | |
| --- | --- | --- | --- | --- | --- |
| **Gene**  **symbol** | **Fold**  **Change** | **Gene**  **symbol** | **Fold**  **change** | **Gene**  **symbol** | **Fold**  **change** |
| USP53 | 8.155 | USP49 | 4.922 | USP53 | 10.842 |
| USP49 | 4.044 | USP22 | 3.473 | USPL1 | 6.187 |
| USP27X | 3.118 | USP53 | 2.807 | USP49 | 4.766 |
| USP25 | 2.568 | USP31 | 2.110 | USP2 | 3.831 |
| USP37 | 2.539 | USP49 | 2.068 | USP25 | 2.926 |
| USP2 | 2.466 | USP3-AS1 | 2.402 | USP48 | 2.406 |
| USPL1 | 2.454 | USP2 | 2.195 | USP37 | 2.384 |
| USP22 | 2.429 | OTUB2 | 2.781 | USP22 | 2.369 |
| USP32/  USP6 | 2.377 | ATXN7L1 | 3.261 | USP42 | 2.086 |
| USP34 | 2.334 | ATXN2L | 2.008 | USP12 | 2.005 |
| USP42 | 2.314 | ATXN8OS | 2.523 | OTUB2 | 3.378 |
| USP25 | 2.216 | ATXN3L | 2.045 | OTULIN | 2.741 |
| USP21 | 2.214 | BAP1 | 2.362 | OTUD4 | 2.195 |
| USP45 | 2.112 |  |  | ATXN3 | 3.703 |
| OTUB2 | 2.281 |  |  | ATXN2L | 2.610 |
| OTULIN | 2.129 |  |  | ATXN1 | 2.592 |
| ATXN7L1 | 2.627 |  |  | ATXN7L1 | 2.116 |
| ATXN3 | 3.543 |  |  | ATXN3L | 2.081 |
| BAP1 | 2.390 |  |  | BAP1 | 2.405 |

**Supplementary Table S2. Signaling pathways governing the osteogenic differentiation of hBMSCs**

| **Ligand** | **Mediator** | **USP53**  **overexpression** | **USP53**  **knockdown** |
| --- | --- | --- | --- |
| **Wnt** | Active-β-catenin | ↑ | ↓ |
| **TGF-β** | Smad2/3 | ↑ | ↓ or ↑ |
| **BMP** | Smad1/5/8 | ↓ | ↑ |
|  | ERK | ↓ | No change or ↓ |
|  | P38 | ↓ | ↓ |
| **PTHrP** | PKA | No change | ↑ |
| **IGF** | AKT | ↓ | ↓ |

**Supplementary Table S3. List of proteins that interact with USP53 (Mass spectrometry)**

| **Band label** | **Number** | **NCBI BLAST** | **Protein name** | **Score** | **Mass** |
| --- | --- | --- | --- | --- | --- |
| **IgG** | 1 | AAH20946.1 | Tubulin, beta  [Homo sapiens] | 1104 | 50096 |
|  | 2 | NP_001060.1 | tubulin beta-2A chain isoform 1 [Homo sapiens] | 905 | 50274 |
|  | 3 | NP_006077.2 | tubulin beta-3 chain isoform 1 [Homo sapiens] | 724 | 50856 |
|  | 4 | CAA25318.1 | tubulin 5-beta  [Homo sapiens] | 637 | 50055 |
|  | 5 | NP_080749.2 | tubulin beta-6 chain  [Mus musculus] | 509 | 50514 |
|  | 6 | EAX01554.1 | tubulin, beta 6, isoform CRA_b, partial  [Homo sapiens] | 353 | 44133 |
|  | 7 | AAH21564.1 | TUBA1B protein  [Homo sapiens] | 287 | 37707 |
|  | 8 | NP_005733.1 | protein disulfide-isomerase A6 isoform d precursor [Homo sapiens] | 187 | 48490 |
|  | 9 | NP_000412.3 | keratin, type I cytoskeletal 10 [Homo sapiens] | 155 | 58994 |
|  | 10 | NP_001677.2 | ATP synthase subunit beta, mitochondrial precursor [Homo sapiens] | 154 | 56525 |
|  | 11 | AAH08633.1 | actin, beta, partial  [Homo sapiens] | 135 | 41321 |
|  | 12 | NP_001395.1 | elongation factor 1-gamma [Homo sapiens] | 94 | 50429 |
|  | 13 | NP_005207.2 | dolichyl-diphosphooligosaccharide—  protein glycosyltransferase 48 kDa subunit precursor [Homo sapiens] | 85 | 50841 |
|  | 14 | NP_006112.3 | keratin, type II cytoskeletal 1 [Homo sapiens] | 84 | 66170 |
|  | 15 | NP_001093.1 | alpha-actinin-1 isoform b [Homo sapiens] | 78 | 103563 |
|  | 16 | 4O33_A | Chain A, Crystal Structure Of Human Pgk1 3pg And  Terazosin(tzn) Ternary Complex | 69 | 44973 |
|  | 17 | NP_001407.1 | eukaryotic initiation factor 4A-I isoform 1 [Homo sapiens] | 58 | 46353 |
|  | 18 | A6NMY6.2 | PUTATIVE PSEUDOGENE: RecName: Full=Putative annexin A2-like protein | 56 | 38806 |
|  | 19 | ALX00019.1 | non-muscle myosin heavy chain 9 [Homo sapiens] | 53 | 227745 |
|  | 20 | AAX82298.1 | actin-like protein, partial [Homo sapiens] | 52 | 11529 |
|  | 21 | ABB04050.1 | rhabdomyosarcoma antigen MU-RMS-40.12  [Homo sapiens] | 49 | 55605 |
|  | 22 | NP_057613.4 | phospholipid-transporting ATPase IB isoform 1  [Homo sapiens] | 46 | 134996 |
|  | 23 | CAA67203.1 | cytokeratin [Homo sapiens] | 45 | 30759 |
|  | 24 | NP_001485.2 | rab GDP dissociation inhibitor beta isoform 1  [Homo sapiens] | 28 | 51087 |
| **Band label** | **Number** | **NCBI BLAST** | **Protein name** | **Score** | **Mass** |
| **In common** | 1 | NP_003371.2 | vimentin [Homo sapiens] | 1165 | 53676 |
|  | 2 | NP_006079.1 | tubulin beta-4B chain  [Homo sapiens] | 881 | 50255 |
|  | 3 | NP_001226.2 | serpin H1 precursor  [Homo sapiens] | 210 | 46525 |
|  | 4 | Q5VTE0.1 | PUTATIVE PSEUDOGENE: RecName: Full=Putative elongation factor 1-alpha-like 3 | 154 | 50495 |
|  | 5 | NP_000691.1 | annexin A1 [Homo sapiens] | 74 | 38918 |
|  | 6 | NP_002129.2 | heterogeneous nuclear ribonucleoprotein D0 isoform c [Homo sapiens] | 66 | 32985 |
|  | 7 | BAS02858.1 | T cell receptor alpha chain V-J-region, partial  [Homo sapiens] | 31 | 7205 |
| **Band label** | **Number** | **NCBI BLAST** | **Protein name** | **Score** | **Mass** |
| **USP53** | 1 | NP_001060.1 | tubulin beta-2A chain isoform 1 [Homo sapiens] | 118 | 50274 |
|  | 2 | Q9BYX7.1 | PUTATIVE PSEUDOGENE: RecName: Full=Putative beta-actin-like protein 3 | 64 | 42331 |
|  | 3 | P0DOX5.2 | RecName: Full=Immunoglobulin gamma-1 heavy chain; | 61 | 49925 |
|  | 4 | BAC02397.1 | immunoglobulin heavy chain VHDJ region, partial [Homo sapiens] | 57 | 13571 |
|  | 5 | AIL30988.1 | immunoglobulin heavy chain variable region, partial [Homo sapiens] | 48 | 12740 |
|  | 6 | AAU50679.1 | F-box protein 31  [Homo sapiens] | 37 | 61408 |
|  | 7 | AGK29038.1 | immunoglobulin heavy chain variable region, partial [Homo sapiens] | 35 | 11800 |

**Supplementary Table S4. Reduced expression of USP53 in the hBMSCs of patients with osteoporosis, as determined by analysis of microarray data**

| **Down-regulated DUBs (*P* < 0.05)**  **(hBMSC of osteoporosis patients 79–94 years old)** | | | |
| --- | --- | --- | --- |
| **Gene**  **symbol** | **Fold**  **change** | **Gene**  **symbol** | **Fold**  **change** |
| DUSP1 | 0.210 | USP10 | 0.577 |
| USP5 | 0.243 | USP36 | 0.577 |
| DUSP27 | 0.270 | DUSP3 | 0.581 |
| USP44 | 0.314 | DUSP6 | 0.586 |
| DUSP13 | 0.371 | DUSP16 | 0.586 |
| DUSP4 | 0.379 | USP35 | 0.613 |
| USP2 | 0.380 | DUSP10 | 0.634 |
| USP29 | 0.384 | LOC100653247///USP22 | 0.645 |
| USP26 | 0.391 | USPL1 | 0.647 |
| USP21 | 0.414 | USP30 | 0.651 |
| DUSP26 | 0.416 | USP39 | 0.665 |
| DUSP15 | 0.438 | USP13 | 0.669 |
| DUSP9 | 0.454 | DUSP7 | 0.720 |
| USP53 | 0.468 | DUSP5 | 0.732 |
| LOC101927562///DUSP8 | 0.481 | USP27X-AS1 | 0.739 |
| USP32///USP6 | 0.501 | USP24 | 0.788 |
| USP30-AS1 | 0.510 | USP18 | 0.793 |
| USP49 | 0.526 | USP11 | 0.870 |
| DUSP18 | 0.547 | USP32 | 0.873 |

**Supplementary Table S5. Primer sequences for qRT-PCR**

| **Gene names** |  | **Sequence (5′ → 3′)** |
| --- | --- | --- |
| ***ACTB*** | Forward | AGAGCTACGAGCTGCCTGAC |
|  | Reverse | AGCACTGTGTTGGCGTACAG |
| ***ALPL*** | Forward | P324388 (Bioneer) |
|  | Reverse |  |
| ***BSP*** | Forward | P188040 (Bioneer) |
|  | Reverse |  |
| ***COL1A1*** | Forward | P157768 (Bioneer) |
|  | Reverse |  |
| ***DLX5*** | Forward | P199945 (Bioneer) |
|  | Reverse |  |
| ***GAPDH*** | Forward | P267613 (Bioneer) |
|  | Reverse |  |
| ***HPRT*** | Forward | CTGGTGAAAAGGACCTCTCGAAG |
|  | Reverse | CCAGTTTCACTAATGACACAAACG |
| ***OCN*** | Forward | AGCAAAGGTGCAGCCTTTGT |
|  | Reverse | CTTCACTACCTCGCTGCCCT |
| ***OPN*** | Forward | CCGTTGCCCAGGACCTGAA |
|  | Reverse | TGTGGCTGTGGGTTTCAGCA |
| ***RUNX2*** | Forward | P229954 (Bioneer) |
|  | Reverse |  |
| ***SP7*** | Forward | P150104 (Bioneer) |
|  | Reverse |  |
| ***USP53*** | Forward | CAGCCAACATTATTGTGCCTTTGC |
|  | Reverse | GGCTGAAAGTGGCATCGAATGC |

**Supplementary Table S6. Details of antibodies used for western blotting**

| **REAGENTS** | **SOURCE** | **IDENTIFIER** | **Dilution** |
| --- | --- | --- | --- |
| **Immunoblot Antibodies** | | | |
| β-Actin | Santa Cruz Biotechnology | sc-47778 | 1:1000 |
| β-CATENIN | Abcam | ab16051 | 1:1000 |
| Active β-CATENIN | Cell Signaling Technology | 4270 | 1:1000 |
| AKT | Cell Signaling Technology | 9272 | 1:1000 |
| ALP | Abcam | ab95462 | 1:1000 |
| Bone sialoprotein | Abcam | ab125227 | 1:1000 |
| COL1A1 | Abcam | ab6577 | 1:1000 |
| DLX5 | Abcam | Ab109737 | 1:1000 |
| ERK1/2 | Santa Cruz Biotechnology | sc-292838 | 1:1000 |
| FBXO31 | Abcam | ab86137 | 1:1000 |
| FLAG | Sigma | F1804 | 1:1000 |
| GAPDH | Santa Cruz Biotechnology | sc-47724 | 1:1000 |
| GFP | Santa Cruz Biotechnology | sc-390394 | 1:1000 |
| GSK-3β | Santa Cruz Biotechnology | sc-9166 | 1:1000 |
| HA | Santa Cruz Biotechnology | sc-805 | 1:1000 |
| HSP90 | Santa Cruz Biotechnology | sc-7947 | 1:1000 |
| Myc | Santa Cruz Biotechnology | sc-40 | 1:1000 |
| Osteopontin | Abcam | ab91655 | 1:1000 |
| p-AKT | Cell Signaling Technology | 9611S | 1:1000 |
| p-ERK | Santa Cruz Biotechnology | sc-7383 | 1:1000 |
| p-GSK-3β | Santa Cruz Biotechnology | sc-11757 | 1:1000 |
| p-P38 | Santa Cruz Biotechnology | sc-7973 | 1:1000 |
| p-PKA | Cell Signaling Technology | T197 | 1:1000 |
| p-SMAD1/5/8 | Milipore | AB3848-1 | 1:1000 |
| p-SMAD2 | Cell Signaling Technology | 3108 | 1:1000 |
| p-SMAD3 | Cell Signaling Technology | 9514 | 1:1000 |
| PKA | Cell Signaling Technology | 4781 | 1:1000 |
| RUNX2 | Millipore | 05-1478 | 1:1000 |
| SKP1 | Cell Signaling Technology | 12248 | 1:1000 |
| SMAD 2/3 | Cell Signaling Technology | 3102 | 1:1000 |
| SMAD1/5/8 | Santa Cruz Biotechnology | sc-6031-R | 1:1000 |
| SP7 | Abcam | ab22552 | 1:1000 |
| USP53 | Sigma | HPA035844 | 1:1000 |
|  | OriGene | TA808887 | 1:1000 |

**Supplementary Table S7. Sequences of shRNAs**

| **ShRNA ID** | **ShRNA target sequence 5’-3’** |
| --- | --- |
| shMOCK | pLKO.1-puro non-target shRNA Control plasmid DNA  (SHC016, Yonsei genome center, Seoul, Korea) |
| shUSP53-1 | GCCACCAAAGAAATATGCTAT  (TRCN0000038839, Yonsei genome center, Seoul, Korea) |
| shUSP53-2 | GCAGCCAAGATTCTAGGGATA  (TRCN0000038840, Yonsei genome center, Korea) |

**Supplementary Table S8. Details of antibodies used for immunohistochemistry**

| **REAGENTS** | **Dilution** | **SOURCE** | **IDENTIFIER** |
| --- | --- | --- | --- |
| Fluorescein isothiocyanate (FITC) | 1:5000 | Santa Cruz Biotechnology | sc-2012 |
| OSTEOCALCIN | 1:100 | Millipore | AB10911 |
| Phycoerythrin (PE) | 1:5000 | Santa Cruz Biotechnology | sc-3738 |
| USP53 | 1:100 | OriGene | TA808887 |
| VIMENTIN | 1:100 | Santa Cruz Biotechnology | sc-373717 |
